# Molecular Mechanisms and New Treatment Strategies for Non-Alcoholic Steatohepatitis (NASH)

**DOI:** 10.3390/ijms15057352

**Published:** 2014-04-29

**Authors:** Akinobu Takaki, Daisuke Kawai, Kazuhide Yamamoto

**Affiliations:** Department of Gastroenterology and Hepatology, Okayama University Graduate School of Medicine, Dentistry and Pharmaceutical Sciences, 2-5-1 Shikata-cho, Kita-ku, Okayama 700-8558, Japan; E-Mails: daicawai@yahoo.co.jp (D.K.); kazuhide@md.okayama-u.ac.jp (K.Y.)

**Keywords:** non-alcoholic fatty liver, non-alcoholic steatohepatitis, microbiome, oxidative stress

## Abstract

Non-alcoholic steatohepatitis (NASH) is a severe form of non-alcoholic fatty liver disease (NAFLD), in which most patients exhibit non-progressive, non-alcoholic fatty liver (NAFL) attributable to simple steatosis. Multiple hits, including genetic differences, fat accumulation, insulin resistance and intestinal microbiota changes, account for the progression of NASH. NAFLD is strongly associated with obesity, which induces adipokine secretion, endoplasmic reticulum (ER) and oxidative stress at the cellular level, which in turn induces hepatic steatosis, inflammation and fibrosis. Among these factors, gut microbiota are acknowledged as having an important role in initiating this multifactorial disease. Oxidative stress is considered to be a key contributor in the progression from NAFL to NASH. Macrophage infiltration is apparent in NAFL and NASH, while T-cell infiltration is apparent in NASH. Although several clinical trials have shown that antioxidative therapy with vitamin E can effectively control hepatitis pathology in the short term, the long-term effects remain obscure and have often proved to be ineffective in many other diseases. Several long-term antioxidant protocols have failed to reduce mortality. New treatment modalities that incorporate current understanding of NAFLD molecular pathogenesis must be considered.

## 1. Introduction

**Figure 1.**
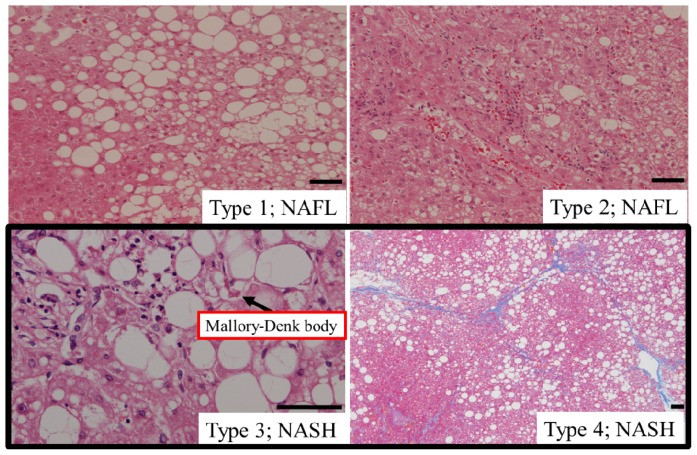
Histological findings of NAFLD. Liver biopsy specimens of NAFLD patients are shown. Representative findings of four classifications are shown, as reported by Matteoni *et al.* in 1999 [2]. **Type 1**: steatosis alone (×100, Hematoxylin and eosin (H and E) staining); **Type 2**: steatosis with lobular inflammation (×100, H and E staining); **Type 3**: steatosis with hepatocyte ballooning (×200, H and E staining) and Mallory-Denk body; **Type 4**: type 3 plus either Mallory-Denk bodies or fibrosis (×40, Azan staining). Scale bar: 50 μm.

Non-alcoholic fatty liver disease (NAFLD) is a common cause of chronic liver disease that is representative of the increasing prevalence of metabolic syndrome [1]. Most patients with NAFLD exhibit non-progressive simple fatty liver, namely non-alcoholic fatty liver (NAFL). Non-alcoholic steatohepatitis (NASH) is a more severe form of NAFLD that is broadly defined by the presence of steatosis with inflammation and progressive fibrosis that ultimately leads to cirrhosis and hepatocellular carcinoma (HCC) (Figure 1) [2–7]. Importantly, a subset of patients with NAFLD develops NASH through poorly understood mechanisms.

**Figure 2.**
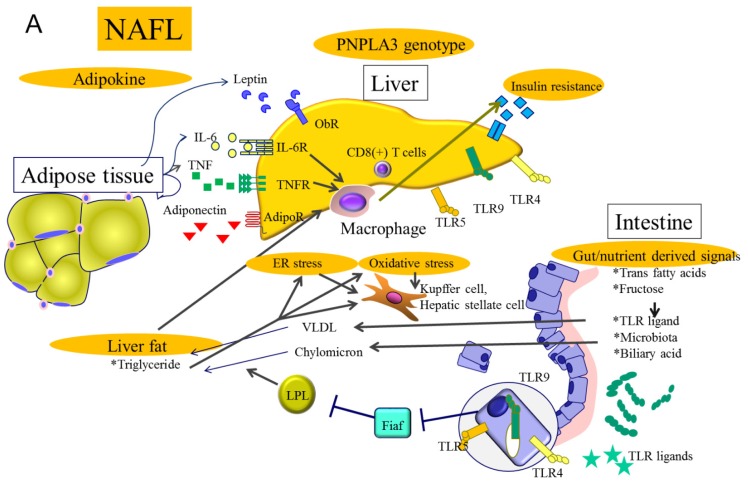

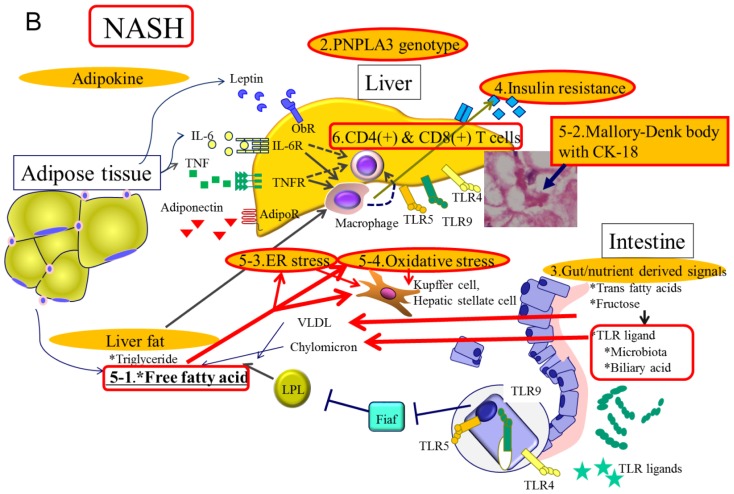
Multiple hits in NAFL and NASH. (**A**) Multiple hits in NAFL are shown. Genome-wide association studies have confirmed the importance of the patatin-like phospholipase 3 (PNPLA3) gene polymorphism in NAFLD. This genetic polymorphism is able to differentiate simple steatosis with or without minimal inflammation and fibrosis progressing to NASH. The gut microbiome has recently been recognized as being involved in NAFLD development. The pattern of microbiome diversity can induce intestinal mucosal permeability and result in lipopolysaccharidemia, which correlates with NASH progression. Enteric bacteria suppress the synthesis of fasting-induced adipocyte factor (Fiaf) resulting in increased lipoprotein lipase (LPL) activity and increased triglyceride accumulation. Obesity and diabetes induce insulin resistance, adipocyte proliferation and changes in intestinal flora. Macrophages play an important role in the induction of inflammation and insulin resistance; (**B**) Ingestion of free fatty acids and free cholesterol induce ER stress and oxidative stress, resulting in hepatic inflammation and fibrogenesis that induces progression to NASH. In some instances, inflammation could precede steatosis, and antitumor necrosis factor (TNF)-α antibody improves steatosis in ob/ob mice. CD4(+)T cells are found after NASH development. Adipokines such as IL-6 and TNF-α produced by adipocytes affect hepatocyte fat content and the liver inflammatory environment. Mallory-Denk bodies are large eosinophilic hepatocellular cytoplasmic protein aggregates containing CK (cytokeratin)-18, which is a hallmark of alcoholic hepatitis and NASH. Heading numbers are consistent with those used in the main text.

The pathophysiology of NASH has been considered a “two hit” process [8]. The first hit is the development of hepatic steatosis via accumulation of triglycerides in hepatocytes, while the second hit includes a variety of cellular stresses, such as oxidative stress, apoptosis and gut-derived stimulation. However, a recent genome-wide association study (GWAS) identified patatin-like phospholipase 3 (PNPLA3) as a key gene in the development of NASH. The involvement of PNPLA3 in NASH pathogenesis indicates that a simple two hit process is insufficient to explain this heterogeneous disease. A “multiple hit” theory has recently been promoted to accommodate the knowledge that inflammation is known to induce steatosis and genetic background is correlated with disease progression (Figure 2).

Although NAFL is usually non-progressive, it can progress in patients harboring the risk allele of the PNPLA3 gene. Increasing evidence has suggested the presence of correlations between intestinal microbiota, bacterial translocation and NAFLD incidence. Alterations in intestinal microbiota resulting from a high-fat diet can induce NASH and exacerbation of related HCC [9].

Obesity and insulin resistance have been accepted as risk factors for NAFLD and NASH progression. Visceral fat induces production of several fat-associated cytokines and induces inflammation, even in patients harboring the non-risk allele of PNPLA3.

Hepatic steatosis is the common feature of NAFLD from early stages to advanced NASH-cirrhosis. Hepatic fat induces cellular stresses and inflammation directly to hepatocytes and also to surrounding non-parenchymal cells. Oxidative stress appears to be responsible for initiating necroinflammation, and reactive oxygen species (ROS), which are generated during free fatty acid metabolism in microsomes, peroxisomes and mitochondria, comprise an established source of oxidative stress [10]. As mitochondria make up the most important cellular source of ROS, mitochondrial dysfunction may play a central role in the progression of NASH.

Immune reactions contribute to one side of hepatitis in NASH. Even in NAFL liver, several inflammatory cell types are present, and these are believed to affect the progression of NAFL to NASH. Gut microbiota-related stimulants and hepatic lipids induce immune reactions.

Several studies have suggested that antioxidants such as vitamin E, along with 1-aminobenzotriazole (ABT), confer benefits upon NAFLD patients, and the American Association for the Study of Liver Disease (AASLD) recommends the use of high-dose vitamin E for NASH [11]. However, most clinical studies involving the treatment of atherosclerotic diseases with dietary antioxidants have not generated clear results, partly because of the non-selective effects of these antioxidative drugs and difficulties associated with cytosolic distribution [12]. The clinical findings of antioxidant therapies have not always been favorable and are often associated with worsening pathology [13]. Thus, new treatment strategies are needed.

Here, we review the current understanding of NAFLD molecular pathogenesis and emerging treatments to be considered in future therapeutic paradigms.

## 2. Molecular Mechanisms Related to Genetic Background in NAFLD

Genetic background, visceral obesity, insulin resistance and hepatic steatosis are features of NASH. A genome-wide association study (GWAS) has reported that a PNPLA3 gene polymorphism is associated with NAFLD susceptibility [14]. The risk allele for this gene was correlated with overall NASH risk, though not so much for obesity and obesity related NASH.

The function of PNPLA3 is unclear, and is complicated by the observation that mice deficient in PNPLA3 develop neither fatty liver nor liver injury. However, overexpression of sterol-regulated binding protein 1c (SREBP-1c), which binds to the transcription start site of the mouse PNPLA3 gene and activates its expression, while PNPLA3 knockdown decreases the intracellular triglyceride content in primary hepatocytes [15]. Thus, PNPLA3 may function as a downstream target gene of SREBP-1c that mediates SREBP-1c stimulation of lipid accumulation. Transgenic mice that overexpress the NAFLD-sensitive PNPLA3 mutant (I148M) in liver, but not in adipose tissue, express altered levels and composition of hepatic triglycerides [16]. Therefore, this risk allele must be the “first hit” to induce hepatic triglyceride accumulation, with subsequent hits needed to affect disease progression.

This genetic variation differentiates between simple steatosis with or without minimal inflammation and fibrosis that progresses to NASH [17,18]. Patients with the NASH-sensitive single nucleotide polymorphism (SNP) rs738409 G/G genotype may progress to simple steatosis as well as to NASH, probably under the same metabolic conditions. This allele results in the I148M variant of the PNPLA3 protein. This allele is also susceptible to hepatocellular carcinoma and poor prognosis [19]. A meta-analysis revealed that this variant is associated with increased liver fat content, when compared to weight-matched individuals not harboring the PNPLA2 polymorphism, and an increased risk of severe fibrosis, even in the presence of other etiologies of chronic liver diseases [20]. However, hepatitis C virus (HCV)-related steatosis may not be affected by polymorphisms, as HCV itself can induce hepatic lipid accumulation [21,22]. Not all patients with progressive NASH have the PNPLA3 risk allele; thus, differences in the characteristics of PNPLA3 risk allele bearing- and non-bearing-NAFLD patients have been demonstrated [23]. PNPLA3-related NAFLD is not characterized by features typical of metabolic syndrome, including hyperinsulinemia, hypertriglyceridemia and low HDL-cholesterol levels [24]. Obesity-related NAFLD patients exhibit the same distribution of PNPLA3 genotype as non-obese patients, whereas inflammation-related genes are upregulated in adipose tissue. Mutant PNPLA3 and wild-type NAFLD showed the same levels of adipocyte inflammatory gene expression, while hepatitis-related pathologies were significantly higher in PNPLA3 mutant patients.

## 3. Molecular Mechanisms Related to Gut Derived Signals in NAFLD

The importance of gut function and microbiota changes on general health is now commonly accepted. Gut microbiota play many roles in NAFLD and NASH, hepatocellular carcinoma, cardiac function, vascular atherosclerosis, diabetes and other conditions. However, gut microbiota patterns are strongly affected by numerous environmental features and the results are often controversial.

Endotoxin or LPS produced by gut microbiota can be delivered to the liver via the portal vein, which raises the question of why such toxic materials are allowed to flow through the intestinal barrier and into the portal vein.

Microbiota changes can induce increases in intestinal permeability that result in inflammatory signal propagation into portal blood and the liver. Altered intestinal permeability in murine models can permit a variety of cellular stresses, such as oxidative stress, endoplasmic reticulum (ER) stress and gut-derived LPS, to induce and trigger inflammatory responses and progressive liver damage [25]. Murine NAFLD models of bacterial overgrowth develop compositional changes and increased intestinal permeability with a concurrent reduction in the expression of tight junction proteins [26]. Plasma endotoxin levels are significantly higher in patients with NAFLD and in murine NASH models [27,28]. High-fat diets are capable of increasing LPS concentrations by two- to three-fold [29]. The intestinal microbiota of diabetic mice can be altered with prebiotic supplementation to increase *Bifidobacterium*, *Lactobacillus* and *Clostridium coccoides*, thereby producing lower plasma LPS and cytokine levels, and decreased hepatic expression of inflammatory and oxidative stress markers [30].

Patients with biopsy-proven NAFLD have increased intestinal permeability with disrupted intercellular tight junctions in the intestine [28]. These abnormalities are related to increased bacterial overgrowth in the small intestine. Microbial colonization of the gut begins immediately after delivery. Even the route of delivery (vaginal or Caesarean section) can affect gut microbiota [31]. Many factors can alter the gut microbiota, including breastfeeding or formula feeding, illnesses, dietary changes, and antibiotic treatment. The composition of intestinal microbiota differs significantly between children in rural areas and industrialized countries. Increased consumption of fruits and vegetables in high-fiber diets and energy-restricted diets prevalent in rural areas are associated with greater amounts of *Prevotella*, lower amounts of *Bacteroides* and increased bacterial gene richness, leading to a lower prevalence of obesity, insulin resistance and inflammatory disorders. However, the underlying mechanisms by which intestinal microbiome patterns affect NAFLD have not been clearly defined. In human NAFLD stool, *Clostridium coccoides* has been shown to be higher in NASH, while *Bacteroides*/*Prevotella* is lower in NASH [32]. Enteric bacteria suppress the synthesis of fasting-induced adipocyte factor (Fiaf), resulting in increased activity of lipoprotein lipase (LPL) and increased triglyceride accumulation in the liver, which indicates a direct influence of the microbiome on NAFLD [33].

Proinflammatory inflammasomes induce hepatic inflammation in patients with NAFLD. However, an inflammasome-deficient mouse model developed exacerbated hepatic steatosis and inflammation through the influx of TLR4 and TLR9 agonists into the portal vein [34]. This strongly supports the hypothesis that the intestinal environment is more important than local hepatic inflammation. The microbiota of these inflammasome-deficient mice differed from those of wild-type mice with NASH. Furthermore, co-housing inflammasome-deficient and wild-type mice resulted in intestinal inflammation and exacerbated hepatic steatosis in wild-type mice. This finding suggests that altered microbiota in inflammasome-deficient mice could be transferred to healthy mice resulting in intestinal inflammation, increased permeability and NAFLD.

Gut and oral periodontal status correlates with the progression of chronic liver disease into HCC [35]. Treating periodontitis could improve transaminases in NAFLD and, in fact, several probiotics that control gut microbiota improve NAFLD [36,37].

Studies using models of hepatocarcinogenesis have found that high-fat diet increases levels of deoxycholic acid, a gut bacterial metabolite that damages DNA and exacerbates hepatocarcinogenesis [9]. Antibiotics are capable of abrogating these effects. Gut microbiota affect not only NAFLD, but also obesity-related hepatocarcinogenesis. Even a short duration of antibiotic treatment results in microbiota changes that can increase the risk of antibiotic resistance and the transfer of resistance genes to pathogens. Therefore, another approach to controlling gut microbiota must be considered. A potential method for the maintenance of the gut microbiota involves the usage of prebiotics and probiotics [38].

## 4. Molecular Mechanisms Related to Obesity and Insulin Resistance in NAFLD

Obesity, hypertriglyceridemia and hypertension are predictive risk factors for NAFLD [39]. Visceral fat accumulation in obesity correlates with various organ pathologies including cerebrovascular diseases, cancer and NASH. Visceral fat accumulation is regarded as a significant risk factor for the development of NAFLD and progressive NASH. However, PNPLA3-related NASH could be seen in lean patients; thus, obesity-related visceral fat accumulation can partially explain NAFLD pathogenesis.

The severity of hepatic steatosis determined by ultrasound positively correlates with visceral fat accumulation and insulin resistance in both obese and non-obese individuals, suggesting that hepatic steatosis is influenced by visceral fat accumulation regardless of obesity [40]. Even among children, visceral obesity has become quite common worldwide over the past decade, thanks to a shift towards Western-style diets that are high in calories, fat and fructose [41]. Such diets and decreased physical activity can also induce loss of muscle mass and strength, referred to as sarcopenia [42]. NASH and sarcopenia share many causative factors and their pathologies are correlated with one another, suggesting a direct effect of physical exercise on NASH.

Visceral obesity induces the inflammatory cytokine interleukin-17 (IL-17) [43]. The IL-17 receptor A is widely expressed in the liver and IL-17 drives neutrophil chemokine expression. Controlling the IL-17 axis has been shown to be effective in treating NASH mouse model progression [44]. In human NASH, neutrophil infiltration has been suggested to be involved in NAFLD progression. Therefore, obesity-related NAFLD must be affected by visceral adipocytes that produce such cytokines.

Insulin resistance is an independent risk factor for NAFLD severity [40]. Adipose and hepatic insulin resistance progressively increases across NAFLD stages even in non-obese, non-diabetic and normolipidemic patients. The oral glucose tolerance test (OGTT) shows impaired pancreatic β-cell function in patients with NASH, but not in those with simple steatosis [45]. Incretins, glucagon-like peptide-1 (GLP-1) and glucose-dependent insulinotropic polypeptide (GIP) are gastrointestinal peptide hormones that regulate postprandial insulin release from pancreatic β-cells. GLP-1 is released from endocrine L-cells into the splanchnic and portal veins and GLP-1 receptor is expressed on human hepatocytes. GLP-1 lowers plasma glucose, improves insulin sensitivity with postprandial insulin release, reduces glucagon secretion and delays gastric emptying [46]. In NAFLD and NASH patients, glucose-induced GLP-1 secretion was significantly decreased when compared with controls, thus suggesting that reduced GLP-1 secretion is involved in NAFLD pathophysiology [47].

Visceral fat accumulation is correlated with increased adipokine levels. The function of lipid droplets was previously viewed simply as an energy storage structure, whereas it is currently considered to be a complex organelle that is involved in many processes including metabolic, immunological and inflammatory responses. Adiponectin is the most abundant adipose tissue-specific “good” adipokine. Adiponectin is mainly produced by adipocytes in mature white adipose tissue, and levels of expression and secretion increase during adipocyte differentiation. Levels of adiponectin are significantly higher in females than in males, in whom serum androgens become more evident during puberty [48]. Adiponectin has anti-inflammatory, antidiabetic and antilipid storing effects, and levels inversely correlate with visceral obesity and insulin resistance. Furthermore, weight loss is an inducer of adiponectin synthesis, whereas proinflammatory adipokines such as TNF-α and IL-6 suppress adiponectin [49]. Adipose tissue is also the main producer of the adipokine leptin, the levels of which directly correlate with body fat mass and adipocyte size [50]. Leptin production is mainly regulated by food intake, and hormones related to eating, such as insulin, increase leptin secretion and *vice versa*. Proinflammatory endotoxin, IL-1 and TNF-α increase the secretion of leptin, which exhibits central and peripheral effects. Peripheral leptin increases basal metabolism, regulates pancreatic cell function and insulin secretion, and affects T-cell generation and differentiation of T helper 1 cells in lymph nodes.

Leptin-deficient (ob/ob) mice and leptin receptor-deficient (db/db) mice are severely obese and have increased pituitary and adrenal hormone production, hyperglycemia, elevated insulin and decreased immune function. Furthermore, db/db mice fed a high-fat diet exhibited reduced adiponectin levels and more than 50% progressed to NASH. This animal model exhibited reduced levels of adiponectin receptor-2 in liver, diminished mitochondrial function and increased inflammation [51].

Adiponectin levels are also an independent predictor of NASH, suggesting the importance of this pathway in NASH development. A meta-analysis of 698 controls and 1545 patients with NAFLD found that serum adiponectin levels are low in NAFLD and much lower in NASH [49]. As adiponectin and leptin exert antagonistic effects on liver fibrogenesis and inflammation, the ratio of adiponectin to leptin may be a better marker for distinguishing NASH from NAFLD. Levels of adiponectin receptor 2 are decreased in human liver biopsy specimens of NASH [52]. However, since results indicating that lower serum adiponectin induces expression of hepatic adiponectin receptor 2 as a compensatory response have been contradicted, the function of these novel adipokines and receptors requires further investigation [53,54].

## 5. Molecular Mechanisms Related to Hepatic Steatosis in NAFLD

### 5.1. Type of Hepatic Steatosis

Both PNPLA3-related and non-related NAFLD exhibit hepatic steatosis, which is a common feature of NAFLD. However, the hepatic fat in NAFL and NASH may have different molecular characteristics.

Triglycerides are the main type of lipid stored in the liver of patients with NAFLD. The toxic lipids in NASH and the non-toxic lipids in NAFL (simple steatosis) may be different [55]. Diacylglycerol acyltransferase 2 (DGAT2) catalyzes the final step in hepatocyte triglyceride biosynthesis. Hepatic steatosis and dietary triglyceride content induced in a model of obese-simple fatty liver are reduced by DGAT2 antisense oligonucleotides in a manner that does not correlate with changes in body weight, adiposity or insulin sensitivity [56]. However, DGAT2 antisense treatment increased levels of hepatic free fatty acids, lipid oxidant stress, lobular necroinflammation and fibrosis in a mouse NASH model, whereas hepatic triglyceride content decreased [55]. These results suggest that the pathogenesis and treatment of steatosis in simple fatty liver and NASH are different.

Analysis of the role of human genetic variability in lifestyle intervention has shown that the DGAT2 gene polymorphism is related to a decrease in liver fat, while changes in insulin resistance are not correlated [57]. As insulin resistance is the key marker for NASH, the DGAT2 gene polymorphism might only be associated with non-progressive fatty liver. As the significance of apparently similar fat droplets in simple fatty liver and NASH hepatocytes differ in DGAT2 knockdown experiments, analyzing the molecular pathogenesis of NASH at the cellular level is important.

### 5.2. Hepatic Steatosis and Cell Death

Many scoring systems to differentiate NAFL and NASH have been reported, including FIB-4 score ( $\text{age}\times \text{AST}/\text{platelet}\times \sqrt{\text{ALT}}$), AST to ALT ratio, BARD score (BMI > 28, AST/ALT > 0.8, diabetes), APRI score (AST to platelet ratio), Fibro test (includes total bilirubin, gamma GTP, alpha 2 macroglobulin, apolipoprotein A1, haptoglobulin), cytokeratin 18 (CK-18) fragments, hyaluronic acid, soluble Fas and Fas ligand, C-reactive protein, fibroblast growth factor-21, adiponectin, and desmosterol [58,59].

Of these, measurement of CK-18 fragments has been shown to be reliable, with more than ten reports from different populations, including children [60,61]. A meta-analysis revealed that plasma CK-18 levels exhibit a sensitivity of 78% and a specificity of 87% [62]. However, one report with 424 middle-aged NAFLD patients exhibited inadequate sensitivity (58%) and specificity (68%) for NASH diagnosis [63]. To date, this is the best marker for diagnosing NASH, but is insufficient to supplant liver biopsy from the diagnostic process.

CK-18 is the major intermediate filament protein in the liver that is released into the extracellular space during cell death. Full length CK-18 can be cleaved by caspase-3, -6 and -7 resulting in 30- and 45-kDa fragments. The 30-kDa fragment can be detected using the M30 antibody. Therefore, soluble CK-18 is believed to be representative of hepatic apoptosis.

High-fat diets are able to induce CK-8/18 accumulation, resulting in Mallory-Denk body formation and hepatocyte apoptosis in the liver [64]. Mallory-Denk bodies are large eosinophilic hepatocellular cytoplasmic protein aggregates containing CK-18, which is a hallmark of alcoholic hepatitis and NASH. The formation of this product of hepatocyte degeneration is induced by saturated fatty acids, which also induces NASH. While the mechanism requires further clarification, several factors are involved including inflammatory cytokine IL-6, cytoplasmic aggregate-related ubiquitin binding protein p62, and reductions in protein misfolding preventive factor HSP72. CK-18-related Mallory-Denk body formation may be correlated with plasma CK-18, which could be a strong marker for differentiating NAFL and NASH.

### 5.3. Hepatic Steatosis and Endoplasmic Reticulum (ER) Stress

Toxic lipids such as free fatty acids, diacylglyceride, phospholipids and free cholesterol, activate several cellular stress pathways [65]. One epicenter for these stress responses is the ER, a membranous network that functions in the synthesis and assembly of secretory and membrane proteins to achieve their proper conformation. Proteins that are related to lipid metabolism are upregulated, whereas protein synthesis and transport functions are downregulated in obese mouse hepatic ER [66]. The maintenance of ER function requires high concentrations of intra-ER Ca^2+^, which is actively controlled by sarco(endo)plasmic reticulum Ca^2+^-ATPase (SERCA). Free cholesterol accumulation triggers ER stress by altering the critical free cholesterol-to-phospholipid ratio of the ER membrane, which is needed to maintain its fluidity. Among the ER enzymes, SERCA ATPase is particularly sensitive to ER membrane cholesterol content, which can inhibit SERCA conformational changes and activity. Such changes induce a decrease in the physiologically high intra-ER Ca^2+^ concentrations that result in impaired ER function known as ER stress. ER stress is one of the most important factors for disease progression in NASH along with hepatocyte apoptosis and hepatic stellate cell (HSC) or Kupffer cell activation.

The chaperone response is blunted in obese mice as a result of dysfunctional X-Box binding protein 1 (XBP1), which is a master regulator of ER folding capacity and key hepatic lipogenic genes [67]. Mice bearing a liver-specific deletion of XBP1 exhibit decreased *de novo* hepatic lipogenesis and decreased steatosis in response to a lipogenic diet [68]. Levels of liver-specific SERCA2b are reduced in obese mice with increased ER stress. Increasing SERCA2b levels reduce ER stress in the liver and increases glucose tolerance [69]. The SERCA inhibitor thapsigargin induces an increase in cytosolic calcium content with a decrease in ER calcium resulting in mitochondrial cytochrome C release and apoptosis of cultured primary rat hepatocytes [70].

Analysis of human liver samples for mRNA and protein expression revealed that XBP1 is overexpressed in NASH but not in NAFL [71]. Kupffer cells function as antigen-presenting cells by displaying major histocompatibility complex (MHC) peptides on their cell surface. These peptides are processed by proteasomal degradation in the cytosol and then translocated to the ER, where they undergo *N*-terminal trimming and loading onto MHC for export to the cell surface. The ER also contains inflammatory cytokine-inducible factors such as stimulator of interferon genes (STING) that induce type I interferon genes upon release [72]. ER stress induces the activation of MHC-related and non-related proinflammatory responses.

These results suggest that diseases can be treated with anti-ER stress agents as noted in the above mouse models. However, ER stress also sensitizes activated, but not quiescent HSC to apoptosis, resulting in the resolution of fibrosis [73]. Non-selective anti-ER stress treatment might be ineffective and treatment targeted towards ER stress should be designed to focus on the specific status of ER conditions.

### 5.4. Hepatic Steatosis and Oxidative Stress

Oxidative stress is involved in the mechanisms of aging, carcinogenesis and atherosclerotic progression. Excessive oxidative stress induced by mitochondrial, peroxisomal and microsomal reactive oxygen species (ROS) in NASH results in apoptosis as well as damage to nuclear and mitochondrial DNA. Limited antioxidant defenses contribute to the processes of both NASH and hepatocarcinogenesis [74,75]. Physiologically low levels of ROS are involved in necessary vital cellular processes indicating that the balance of oxidative-antioxidative responses is important [76]. Mitochondrial dysfunction not only impairs fat homeostasis in the liver but also leads to an overproduction of oxidative stress, resulting in the generation of reactive oxygen species (ROS) that trigger lipid peroxidation, cytokine overproduction and cell death. Indeed, ultrastructural alterations, impairment of ATP synthesis and increased production of ROS have been reported in liver mitochondria from NASH patients as well as in a rodent model [77,78]. Mitochondria are a principal source of cellular ROS due to inefficiencies in electron flow along the electron transport chain (ETC). Under physiological conditions, the majority of incompletely reduced ROS, such as superoxide, are detoxified into water and steady-state oxidant concentrations are maintained at relatively low levels (less than 1% of total oxygen consumed by mitochondria) by a variety of antioxidant defenses and repair enzymes [79]. The mitochondrial capacity to control oxidative balance collapses under continuous oxidative stress. Excess superoxide could be generated within injured mitochondria through electron leakage and the resulting excess of superoxide would be converted to hydrogen peroxide (H_2_O_2_) by superoxide dismutase (SOD). Glutathione peroxidase (GPx) or catalase can metabolize H_2_O_2_ to non-toxic H_2_O, but the Fenton and/or Haber–Weiss reactions generate the highly reactive and toxic hydroxyl radical.

Iron is the key mineral that induces oxidative stress produced via the Fenton reaction. Although its role in NASH is not fully understood, levels of iron are elevated in NASH, which is an inducer of oxidative stress, and reducing iron levels has resulted in fair outcomes for patients with chronic liver diseases [80]. However, one-third of early-stage NAFLD patients show iron deficiency correlated with female gender, obesity and type 2 diabetes [81]. We must wait for long-term follow-up studies to learn whether iron-deficient obese patients progress to NASH and to understand the role of iron in NAFLD progression *in vitro.*

The mitochondrial proliferation and differentiation program may be impaired in NASH. One of the most important regulators of mitochondrial biogenesis is the transcription coactivator PPAR-γ-coactivator-1α (PGC-1α) [82], which coordinates the large number of genes required for mitochondrial biogenesis. The activity of PGC-1α is impaired in fatty liver, which results in decreased mitochondrial biogenesis [83].

In NASH-related HCC models, PGC-1α was downregulated in HCC when compared with non-tumorous tissues, thus signifying its importance in normal hepatocyte phenotype [84]. Decreases in mitochondrial DNA and mitochondrial DNA-encoded polypeptides are representative findings in NASH, whereas mitochondrial DNA content is increased in simple fatty liver [85]. The complementary activation of mitochondrial DNA in simple fatty liver may help to protect the liver from inflammation and fibrosis, whereas decreases in NASH induce progressive inflammation and fibrosis with normal hepatocyte function disturbance.

The non-parenchymal cells in the liver, such as Kupffer cells and HSC, play significant roles in the progression of chronic liver inflammation and fibrosis progression [86]. Excess fatty acid accumulation in hepatocytes induces oxidative stress from not only mitochondria but also peroxisomes or microsomes. These cytotoxic ROS and lipid peroxidation products are able to diffuse into the extracellular space affecting Kupffer cells and HSC. Cellular oxidative stresses from hepatocytes, and the direct uptake of free fatty acids or free cholesterol in Kupffer cells, induce activation of nuclear-factor κB, which induces synthesis of TNF-α and several proinflammatory cytokines such as IL-6 or IL-8 [87]. Kupffer cells in patients with NASH produce TGF-β, resulting in HSCs acquiring a fibrogenic myofibroblast-like phenotype.

Exposing primary HSC or HSC cell lines to H_2_O_2_ leads to an increase in gene expression of ER chaperone BIP binding transmembrane proteins such as inositol requiring enzyme 1 (IRE1a), or activating transcription factor 4 (ATF4). ER stress in HSCs results in increased autophagy and HSC activation to fibrogenic status [88]. Among the most characteristic features of HSC activation is the loss of cytoplasmic lipid droplets, which are composed of retinyl esters and triglycerides [89].

Autophagy is present in all cell types and is upregulated as an adaptive response under cellular stress to generate intracellular nutrients and energy. Autophagy is upregulated in activated HSC of mice with liver damage. However, cytoplasmic lipid droplets are maintained and remain quiescent in autophagy-defective HSCs, indicating that oxidative stress-induced ER stress and autophagy is a key event in HSC activation [90].

## 6. Molecular Mechanisms Related to Immune Reactions in NAFLD

Immune responses and inflammation are known to be involved in metabolic diseases, such as diabetes mellitus, atherosclerosis and NASH. Adipose tissue-derived cytokines are known to promote metabolic disease progression. In obesity, excess proinflammatory, M1-like macrophages accumulate in adipose tissue and liver [91]. Even in simple fatty liver, macrophage infiltration and expression of the macrophage attractant chemokine CCL2 are significantly increased [92]. Furthermore, inflammatory macrophages induce insulin resistance. However, it is not clear which factors delineate the progression of inflammation under the same obesity conditions.

Inflammatory pathways in macrophages are under strict control by several transcription factors such as NF-kB, AP1, the peroxisome proliferator-activated receptor (PPAR) family, LXR, and associated coactivating and coregulatory molecules. The major corepressor, nuclear receptor corepressor (NCoR), has an important role in metabolic processes. In the basal state, NCoR is resident on a number of inflammatory pathway genes, thereby maintaining them in the repressed state. Upon inflammatory pathway activation, NCoR dissociates from the promoter complex following activation of proinflammatory transcription factors such as NF-kB and AP1 [93]. Conversely, macrophage- and neutrophil-specific NCoR knockout mice display an anti-inflammatory and insulin-sensitive phenotype. This supposed opposite phenotype is due to derepression of LXR, which leads to the induction of the lipogenic pathway and increases the biosynthesis of palmitoleic acid and omega 3 fatty acids, which exert local anti-inflammatory effects and insulin sensitivity [94]. Adaptive immune response-linked CD8 (+) T cells usually respond after innate immune cell reactions; however, obese adipose tissue can recruit CD8 (+) T cells before macrophage accumulation in high-fat-diet-fed mice [95]. Therefore, many types of inflammatory cells can accumulate in obesity which, along with the accompanying increase in adipose tissue, results in inflammation and insulin resistance.

In advanced NASH, CD4 (+) and CD8 (+) T cell infiltration increases and inflammatory cytokines, such as IL-6 or IL-8, are also increased [92]. Such inflammatory T cell infiltration must have important roles in obese mice. Neutrophils are also frequently found in NASH liver specimens and are thought to play important roles. Neutrophils secrete several proteases such as neutrophil elastase. Treatment of hepatocytes with neutrophil elastase causes cellular insulin resistance and the deletion of neutrophil elastase improves insulin resistance and tissue inflammation [96].

Toll-like receptors are sensors of microbial and endogenous danger signals that are expressed and activated in innate immune cells and liver parenchymal cells, and they contribute to the progression of NASH. Gut microbiota might release pathogen- or damage-associated molecular patterns (PAMPs or DAMPs), which are TLR ligands following activation of downstream signals. Ten TLRs (TLR1-10) have been identified in humans and 13 (TLR1-9, 11–13) determined in other mammals. In a mouse fructose-induced NAFLD model, all TLRs tested (1, 2, 3, 4, 5, 6, 7, 8, 9) were upregulated in the liver [97]. These factors may induce both disease progression and disease protective effects. However, gut microbiota are affected by various circumstances and the resulting TLR response may also different.

TLR 2 is a receptor for multiple glycolipids or lipoproteins on the surface of bacteria adhering to the cell surface of monocytes, myeloid dendritic cells or mast cells. With the choline-deficient amino acid-defined (CDAA) diet model mouse, which develops steatosis with relatively mild hepatitis with obesity, TLR2 knockout resulted in improvement of NASH [98]. However, with the methionine and choline-deficient (MCD) diet model mouse, which develops steatosis with severe hepatitis and fibrosis without obesity, TLR2 knockout resulted in progression of NASH [99]. These discrepancies are valid, as these models can exhibit only one facet of NASH. The CDAA diet model is likely to be similar to non-progressive obese NAFL, while the MCD diet is likely to be similar to progressive NASH in malnourished patients.

TLR 4 is an LPS receptor located on the surfaces of monocytes, myeloid dendritic cells, mast cells, B cells, and intestinal epithelium. The interaction of this receptor with representative pathogens of gut microbiota has been studied in detail. Levels of free cholesterol (but not cholesterol ester) are increased in HSC in NAFLD, resulting in increased TLR4 protein levels and fibrogenic HSC [100]. Kupffer cells are phagocytes of various cellular, viral, or bacterial components that are sources of hepatic proinflammatory and profibrogenic cytokines. Phagocytosis of cholesterol by Kupffer cells is able to induce their activation along with TLR4 upregulation [101]. Free cholesterol is able to accumulate in fibrogenic HSCs, resulting in an increase in TLR4 through suppression of the endosomal-lysosomal degradation pathway of TLR4. The increased expression of TLR4 sensitizes cells to TGF-β-induced activation [100].

TLR 9 is located on endoplasmic reticulum (ER) or endosomes of plasmacytoid dendritic cells or B cells and is regarded as a receptor for unmethylated CpG DNA particles that are released from bacteria. These molecules have been analyzed in several NAFLD and NASH models. The TLR4 and TLR9 agonists can flow into the portal veins of inflammasome-deficient MCD mouse models and thus exacerbate NASH [34]. Inflammasomes are multiprotein complexes composed of nucleotide-binding domain and leucine-rich repeat protein 3 (NLRP3), apoptosis-associated speck-like protein containing CARD (ASC), and procaspase 1, which are DAMP or PAMP sensors. Inflammasome activation leads to the processing and secretion of the proinflammatory cytokines IL1β and IL-18, whereas knockdown results in MCD NASH exacerbation. These perplexing findings indicate that the intestinal DAMP or PAMP barrier dysfunction-induced TLR4 or TLR9 overflow overcomes the inflammasome knockout-induced local anti-inflammatory effect in the liver.

TLR 5 is the ligand for microbial flagellin. Mice deficient in TLR-5 develop spontaneous colitis, profound metabolic syndrome including hyperphagia, hypertension, and insulin resistance resulting from intestinal microbiota transport [102,103]. However, another study found no such pathologies in TLR5-deficient mice [104]. The authors concluded that this discrepancy might have been induced by their animal facilities, a factor that might be important to consider for all such molecular function studies.

As immune reactions in NASH are affected by intestinal microbiota and adipocyte status, which are affected by personal lifestyle, it is difficult to postulate definitive explanations.

## 7. Molecular Mechanisms Related to Treatments for NAFLD

### 7.1. General Aspects

Since NAFLD has emerged as a lifestyle-associated disease, lifestyle intervention is an important approach to its treatment. Even a one-year intensive lifestyle intervention comprising dietary modifications and physical activity was able to improve waist circumference, visceral abdominal fat, blood pressure, insulin resistance, and hepatic fat contents in obese patients [105]. Western-style diets, particularly those that are rich in trans-fatty acids, are powerful inducers of obesity and NAFLD, and therefore need to be avoided [106]. However, maintaining compliance with therapeutic measures involving restricted food consumption is emotionally challenging with a high rate of non-compliance [107]. Such patients require pharmacological therapies.

### 7.2. Antioxidant Supplementation as Standard Treatment for NASH

Vitamin E supplementation is a representative antioxidant drug treatment that has become the standard treatment for NASH [108]. The AASLD recommends the use of vitamin E with a daily dose of 800 IU, which is a higher dose than usual [11]. Administration of vitamin E improves non-alcoholic fatty liver disease activity scores (NAS) for clinical and histological activity within two years, but increases insulin resistance and plasma triglyceride levels [109]. However, the recovery of fibrosis progression has not been demonstrated [110]. Antioxidants do not affect body weight, waist circumference, and cholesterol metabolism.

Controversy surrounds antioxidant therapies because ROS have essential functions in living organisms. Antioxidants exhibit effective chemical activity *in vitro*; however, there have been many failures in demonstrating *in vivo* effects [111]. Many cerebrovascular studies have investigated the effects of the vitamin E. A meta-analysis of the effects of vitamin E on stroke revealed a 10% reduction in ischemic stroke accompanied by a 22% increase in hemorrhagic stroke. Antioxidants are likely to contribute to the progression of cancer [112]. Stem cell-like cancer cells have powerful antioxidative properties that protect them from oxidative stress and thus prevent apoptosis [113]. Oxidative stress upon normal cells may induce a transition to a cancer cell phenotype that is highly resistant to further oxidative stress. Inducing oxidative stress under these conditions is an approach that is being investigated as a cancer treatment in several clinical trials [114]. However, this approach is likely to be toxic to normal cells, and may lead to the induction of further carcinogenesis. Thus, oxidative stress must be controlled according to clinical circumstances. The clinical findings of antioxidant therapies have not always been favorable [13]. This might be due to the ineffective uptake of antioxidants into the mitochondria, as well as interference with the essential oxidative stress mechanisms that protect cells from infection or other invasive cellular injuries [115].

### 7.3. Second-Line Treatment Options for NASH

Because the general characteristics of NASH include obesity and insulin resistance, the use of anti-insulin resistance treatment has become prevalent. The PPAR-α agonist pioglitazone and metformin have been clinically shown to improve NASH. Metformin has brought about histological improvements in the non-diabetic NASH mouse model [116]. However, neither of these drugs is capable of improving human NASH liver histology. Metformin induces weight loss, whereas pioglitazone induces weight gain. Human trials are usually too short to generate histological improvements. These antidiabetic insulin-sensitizing drugs also have antioxidative effects.

Glucagon-like peptide-1 (GLP-1) and gastric inhibitory polypeptide (GIP) are both incretins, which are a group of gastrointestinal hormones that cause increased insulin release from pancreatic beta cells, reduce appetite, and might be good targets for treating NASH. A GLP-1 receptor agonist analog improved metabolic, biochemical and histopathological indices of NASH in mice via restoring hepatic lipid oxidation [117]. A GLP-1 receptor agonist in type 2 diabetic patients with NAFLD caused a reduction in intrahepatic lipid content that correlated with diabetic control [118]. GLP-1 agonists (GLP-1 and GIP) are degraded by dipeptidylpeptidase-IV (DPP-IV); therefore, inhibition of DPP-IV extends the half-life of endogenous GLP-1 and GIP and results in diabetic control. The long-term administration of a DPP-IV inhibitor has reduced liver fat content in animals with diet-induced hepatic steatosis and insulin resistance [119].

Pentoxiphylline is a methylxanthine derivative that increases red blood cell flexibility, reduces blood viscosity and decreases platelet aggregation. In addition, pentoxiphylline suppresses TNF-α gene transcription and is a hydroxyl and peroxyl radical scavenger that exhibits antioxidative effects. A randomized controlled trial (RCT) has proven that pentoxiphylline decreases free-radical-mediated lipid oxidation and improves clinical and histological NASH [120,121].

Lipid-lowering drugs such as statins can also improve ALT and radiological steatosis in hyperlipidemic patients with NAFLD; however, histological improvements are not evident [122]. Ezetimibe is a Niemann-Pick C1-like protein inhibitor that can reduce the intestinal accumulation of free cholesterol. Ezetimibe has reduced histological NAS development in mice and in 10 patients with NASH, indicating a need for larger RCT [123,124]. Experiments in mice have revealed that ezetimibe does not improve hepatic steatosis induced by a high-fructose diet, which results from hepatic lipogenesis, yet was able to improve high-fat diet-induced steatosis, which partly results from lipid trafficking via the small intestine [125]. This drug may have favorable effects on NAFLD.

Ursodeoxycholic acid (UDCA) is also reportedly effective in some instances. Several RCTs have found improvements in ALT but not in liver histology, even at high doses [126]. While combinations of UDCA and vitamin E have not been confirmed to be effective, they have improved ALT and histological NAS scores and might be adopted in next-generation treatment protocols [127].

Tumor necrosis factor (TNF)-α is one of the main cytokines involved in adipocyte-related inflammation. Powerful anti-TNF-α agents such as infliximab (chimeric monoclonal antibody), adalimumab (a human monoclonal antibody) and etanercept (fusion protein) have severe side effects, such as tuberculosis, that would render them unacceptable in therapy for NAFLD [128]. The antioxidative agent pentoxifylline also has an anti-TNF-α function that is partially involved in mediating the favorable effects on NASH.

A proinflammatory intestinal microbiome has been identified in mice and in patients with NASH. Probiotics such as butyrate-producing agents reduce hepatic triglyceride contents and induce antioxidative enzymes that help to prevent the progression of NASH to hepatocellular carcinoma [129].

### 7.4. Emerging Treatment Candidates for NASH

#### 7.4.1. Anti-Obesity Medication

Several anti-obesity medications are commercially available, including: (1) inhibitors of fat absorption; (2) inhibitors of the endocannabinoid system; and (3) modifiers of central nervous system neurotransmission involving norepinephrine, dopamine and serotonin [130]. Pancreatic lipase inhibitors, such as Orlistat or Cetilistat, bind to lipase in the gut lumen and prevent the hydrolysis and metabolism of dietary fat (triglyceride), thereby reducing its absorption. A meta-analysis revealed that 26% of those given Orlistat lost ≥10% of total body weight and 30% of this group lost ≥5%, resulting in a 37.3% reduction in the risk of developing type 2 diabetes. While this drug is likely to be effective in NAFLD, it produces adverse effects such as fatty stool-related fecal incontinence and abdominal discomfort. Additionally, its use results in the malabsorption of fat-soluble vitamins such as vitamin A, D, E and K. Longer follow-up studies are necessary in order to establish the long-term life-saving effects of this drug.

#### 7.4.2. New Antioxidants

l-Carnitine is a precursor of carnitine-palmitoyltransferase 1 (CPT-1), the rate-limiting enzyme for mitochondrial β-oxidation that is central to mitochondrial function. Deficiencies in the mitochondrial carnitine-dependent transport system result in curtailed fatty acid oxidation. l-Carnitine is not simply an inhibitor of oxidative stress that must be preserved in certain conditions, but also has the ability to reverse mitochondrial function. l-Carnitine supplementation reduces TNF-α, liver function parameters, plasma glucose levels and histological scores [131]. Furthermore, l-Carnitine ameliorated fatty liver in high-calorie diet/streptozotocin-induced type 2 diabetic mice by improving mitochondrial function [132].

Dietary intake of tomatoes has been reported to reduce the risk for human cancers [133]. Tomatoes include vitamins A and C, and phytochemicals such as carotenoids or flavonoids. Lycopene is the most abundant carotenoid found in tomato, tomato products, and other red fruits. Intake of lycopene inhibits NASH-promoted rat hepatic preneoplastic lesions [134]. Fresh tomato includes 9-oxo-10,12-octadecadienoic acid (9-oxo-ODA), which acts as a PPARα agonist to improve NASH in mice. In addition, processed tomato products such as tomato juice—but not fresh tomato—contains 13-oxo-9,11-octadecadenoic acid (13-oxo-ODA), an isomer of 9-oxo-ODA that improves lipid and carbohydrate metabolism disorders in NAFLD model mice [135]. Although clinical trials remain necessary, plasma carotenoid levels in NASH patients have been shown to be low, thus suggesting the possibility of a role for tomato intake in NASH prevention.

Molecular hydrogen has been shown to have powerful antioxidant effects with unique features [136]. In cultured cells, hydrogen scavenges hydroxyl radicals, but not superoxide, hydrogen peroxide (H_2_O_2_) or nitric oxide (NO), and prevents the decline in the mitochondrial membrane potential and the subsequent decrease in cellular ATP synthesis, consistent with antioxidative effects. Most hydrophilic compounds are retained at membranes and fail to reach the cytoplasm, whereas hydrophobic compounds such as vitamin E cannot penetrate biomembranes in the absence of specific carriers or receptors. In contrast, H_2_ can freely diffuse into cytoplasm and intracellular organelles such as the mitochondria, ER and nucleus. The effects of hydrogen on chemically induced liver damage have been studied in mouse models of liver damage induced by GalN/LPS, CCl_4_ and diethylnitrosamine (DEN) [137]. Hydrogen was given intraperitoneally every three hours after administration of these chemicals. Serum levels of TNF-α and IL-6, as well as transaminase levels, decreased in mice with GalN/LPS-induced acute liver injury after H_2_ administration. Hepatic fibrogenesis markers such as collagen-α1 or α-smooth muscle actin (SMA) were reduced in mice with CCl_4_-induced liver cirrhosis and hepatocyte proliferation was reduced in mice with DEN-induced hepatic tumorigenesis.

Kawai *et al.* reported that drinking hydrogen-rich water has favorable effects in NASH models [74]. Plasma transaminase levels, histological NAS, hepatic TNF-α, IL-6 and fatty acid synthesis-related gene expression and the oxidative stress biomarker 8-OHdG were decreased in the livers of established MCD diet-induced NASH models administered hydrogen-rich water or the antioxidant pioglitazone. Although the decrease in hepatic cholesterol was smaller in the group given hydrogen-rich water, serum oxidative stress was reduced and antioxidant function was higher than in the pioglitazone group. Hydrogen administration also induced a reduction in hepatocarcinogenesis in the NASH HCC mouse model.

As mentioned above, new antioxidants may be effective in controlling NAFLD progression. However, many investigations into the effects of antioxidants upon diseases associated with oxidative stress have been disappointing, and the effects of the newer antioxidants could potentially be the same as those of the previous discouraging agents. More basic and clinical experimentation into this novel potential treatment option is required.

#### 7.4.3. ER Stress-Targeted Treatment

Cellular protein folding-related chaperones are involved in ER stress. The chemical chaperone UDCA has produced relatively disappointing data, as mentioned above. However, UDCA treatment has the potential to improve NAFLD as several markers were improved following its utilization. Other chemical chaperones, such as 4-phenylbutyric acid, have been shown to reduce ER stress by facilitating proper protein folding and trafficking resulting in fatty degeneration in a type 2 diabetes model [138]. Similar to oxidative stress, ER stress is a cell survival response and requires extensive experimental analysis to elucidate the potency and timing of ER stress targeted drug administration.

#### 7.4.4. Adiponectin Receptor Agonist

The adiponectin receptor agonist AdipoRon is considered to be a new treatment candidate for type 2 diabetes [139]. AdipoRon was identified by screening small molecules in a chemical library, and has the ability to bind both adiponectin receptors 1 and 2 (AdipoR1 and AdipoR2) and increase AMPK activation, PGC-1α expression and mitochondrial biogenesis. Administration of AdipoRon induced phosphorylation of AMPK in muscle and liver of wild-type mice but not Adipor1^−/−^Adipor2^−/−^ mice, indicating that this small molecule activates AMPK in skeletal muscle and liver via AdipoR1 and AdipoR2. Orally administered AdipoRon resulted in significantly reduced fasting plasma glucose and insulin levels as well as glucose and insulin responses during oral glucose tolerant tests. Plasma concentrations of triglycerides and free fatty acid (FFA) were also reduced following AdipoRon administration. In skeletal muscles, AdipoRon increased the expression of genes involved in mitochondrial biogenesis and increased mitochondrial DNA contents. In the liver, AdipoRon reduced triglyceride contents, oxidative stress, and inflammatory cytokine expression, indicating that this is likely to be effective in NASH.

## 8. Conclusions

Differentiating non-progressive NAFL and progressive NASH is a fundamentally important issue that remains to be resolved. The apoptosis-related marker CK18 may be a good candidate marker for clinical assessment. The molecular mechanisms of this lifestyle-related disease, which has a genetic component, are complex and the treatment strategies are similarly complex. Although vitamin E administration is the only treatment known to be effective, longer patient follow-ups are necessary as many studies have indicated that caution is required to avoid the potential life-threatening effects of antioxidants. Optimal treatment protocols and combinations of these therapies warrant further investigation.
